# Psychiatry during National Socialism: Contacts with relatives of the victims of NS-Euthanasia as part of a consequent Memorial Culture

**DOI:** 10.1177/0957154X251318501

**Published:** 2025-03-30

**Authors:** Herwig Oberlerchner, Helge Stromberger

**Affiliations:** Institute of Psychology, University of the Alpe Adria Klagenfurt, Austria; Department of Psychiatry and Psychotherapy, Klagenfurt Clinical Center at Wörthersee, Austria; Researcher, Austria

**Keywords:** Psychiatry, National Socialism, euthanasia, compulsive sterilization, commemorative culture, trauma therapy, collective trauma, transgenerational transmission

## Abstract

Crimes against humanity took place in many institutions as well as psychiatric departments during the Nazi dictatorship. The cruel and inhumane history of psychiatry during National Socialism is already widely known and broadly published. Victims of the Nazi regime, of the Holocaust, of racism, racial hygiene, and hereditary biology have often been the subjects of scientific inquiry; the relatives and descendants of the killed inpatients have yet to be part of a scientific or psychotherapeutic response. Analogous to the psychiatric–psychotherapeutic approach with traumatized individuals, the procedure “secure—describe and work through—reconnect” was used in collective remembrance work and commemorative culture. As an example of one part of this collective memorial process, the grief work undertaken with the relatives of the victims of Nazi crime is presented. A phenomenological description presents the results of contact with 55 relatives and offspring of victims of Nazi crimes in psychiatric or associated institutions during National Socialism.

## Foreword

The inhuman happenings within psychiatric departments during National Socialism must not be forgotten. Memory and grief work is constantly necessary in order to keep watch and to warn against it ever happening again. Integration instead of exclusion, protection instead of eradication, high regard even for the weak and foreign, acceptance instead of correction and great respect for life are principles of every modern culture and must especially be lived consciously and proactively by psychiatric departments (Oberlerchner, 2011).

These are some of the important lessons we have to learn from Nazi psychiatry (von Cranach, 2010). The author certainly disassociates himself from the inhumane rhetoric of National Socialism, and he uses quotations and “professional terminology” only to also make transparent the inhuman attitude veiled in the language of the time.

## Introduction

Under National Socialism, psychiatrists showed contempt towards their patients in their care; they lied to them, and deceived them and their families. They forced them to be sterilised, arranged their deaths and even performed killing themselves. Patients were used as test subjects for unjustifiable research – research that left them traumatized or even dead (Schneider, 2011).

Thus begins the speech of Frank Schneider, at the time president of the German Psychiatric Association, on the occasion of a commemoration ceremony on November 26, 2010. The speech culminated in his asking the victims and their relatives for forgiveness. Doctors and psychiatrists planned and participated in the killing of more than 200,000 psychiatric patients. Christian Haring, then president of the Austrian Society for Psychiatry and Psychotherapy (ÖGPP), made a similar statement at the ÖGPP’s annual meeting in 2013. Indeed, the request for forgiveness for the long silence, the trivialization and suppression, was a necessary and important signal, but it is also important to proactively embrace a consistent culture of remembrance in those institutions involved in the crimes.

## Background: Klagenfurt psychiatry under National Socialism

It took several years before the state parliament resolved to establish the “state insane asylum” in the city of Klagenfurt in November 1877. After erecting the new buildings and undertaking several renovations, the highest number of patients was reached at the beginning of the year 1940 with 864 patients (Stromberger, 2003).

The health and political science of the [National Socialist] regime aimed to support those who could contribute to the health and competitiveness of the national economy and the “body of the folk” (*Volkskörper*). By contrast, those who could endanger the efficiency of the economic effort and the health of the collective body are identified and eliminated (Roelke, 2010).

These “efforts” in the interest of the “health of the folk” and the “protection” of German racial ethnicity culminated in a euthanasia program for the “incurably ill,” the direct “eradication” of unwanted ethnicities and undesirable sick people, and mass sterilization (Mitscherlich and Mielke, 2004).

The “Law for the Prevention of Hereditarily Diseased Offspring” came into force on January 1, 1940, in Austria. The law states: “Whoever has a hereditary disease, can be made infertile by a surgical procedure (sterilized), if, according to medical science, a high probability exists that his descendants will suffer a serious physical or mental defect” (Nissen, 2005). To these “inherited diseases” were added schizophrenia and bipolar disorder (manic-depressive illness). In the Austrian state of Carinthia, at least 568 sterilizations are believe to have been performed (Oberlerchner and Stromberger, 2014).

The inhumane attitude of the National Socialist regime increased even further. The Main Office II of the Chancellery of Adolf Hitler on Tiergartenstrasse 4 in Berlin established a special department that, as of 1939, organized the killing of the mentally ill (*Aktion T4*). Reporting forms were sent to the health and nursing asylums, and “experts” and commissions divided patients up into those whose lives were deemed worth living and those whose lives were deemed unworthy of living (*lebenswertes und lebensunwertes Leben*). Reports were required about all unproductive patients who suffered from certain diseases (schizophrenia, epilepsy, senility, intractable paralysis, all-cause imbecility, encephalitis, Huntington’s disease, and other neurological end states) or who had been in the institution continuously for at least 5 years, retained as criminally insane, or who did not have German citizenship or were not of German or kindred blood (Roelke, 2010). In May 1940, one of these commissions comprising five doctors and typists evaluated the departments of the Klagenfurt Regional Hospital, inspected the medical files, and, drawing on other experts from a group of 40 doctors in Berlin, “worked through” a list of names of people to be sent to other institutions of the “*Altreich*,” in order to relieve the strain on the overcrowded department, under the pretext of creating room for new admissions. The transports took place on June 29 and August 25, 1940, and on March 24 and July 7, 1941. The destination of the transports was Hartheim Castle near Linz, where the patients were gassed soon after arrival. At least 739 persons were sent from Klagenfurt to Hartheim.

After protests from relatives, from personnel at the institutions concerned, and involvement from the church, including a rapidly disseminated sermon by the Bishop of Galen on August 3, 1941, the regime decided to decentralize the killing machinery. People were now killed in Klagenfurt. High doses of morphine, but mostly barbiturates like Luminal or Veronal, were administered to persons in the “infirmary” and/or geriatric wing. People thus died of the direct effects of poisoning or indirectly by aspiration and pneumonia and starvation. Dr. Niedermoser and some nurses selected and murdered between 700 and 900 persons from 1942 to 1945.

## Memorial Culture

Analogous to the psychiatric–psychotherapeutic approach with traumatized individuals, the procedure “secure—describe and work through—reconnect” was used to deal with the collective trauma within the framework of remembrance work, and this commemorative culture has been lived since the 1980s in Carinthia (Oberlerchner and Stromberger, 2022).

1. Secure: Securing the memory, mourning culture, commemoration, securing documents and medical records, erecting memorials and places of remembrance;
2. Describe: Publications, public relations, education and lessons, tours to the locations of the crimes;
3. Work through: Working with relatives using detailed descriptions of the care of the 55 families so far;
4. Reconnect: Further development of the discipline of psychiatry by taking a stand on relevant topics such as assisted suicide or strengthening professional ethics (von Cranach, 2010).

## Talks with relatives of the victims as part of a consequent Memorial Culture

As a result of the sometimes very high-profile media coverage and prolific publication activity, several families contacted the Department of Psychiatry in Klagenfurt in order to obtain clarity about the fate of their relatives.

Through an article in the *Kleine Zeitung* [a local newspaper] I was reminded of the fate of my grandmother. She came to the psychiatric ward as a handicapped person, was also said to have had a child. She was never talked about, but everyone suspected a terrible crime.

Research on the family chronicle leads me to you. Gradually, with the help of committed people, I am shedding light on the darkness surrounding my grandfather’s brother that has existed for decades.

A first survey and preview of the work with relatives and descendants of victims of sterilization, deportation, and murder during National Socialism was published in 2015 (Oberlerchner and Stromberger, 2015). There, an initial attempt was made to characterize those people who had contacted the author and his team between 2010 and 2014. Nineteen cases were included. Between December 2014 and July 2023, a further 36 inquiries were registered and processed.

The following short, phenomenological description presents the results of the contact with the 55 relatives. Personal meetings and conversations with relatives were held in 38 cases, involving between one and six family members at a time. In the other cases, communication was by mail or over the phone. In eight cases, it was not possible to confirm the fate of the persons inquired about, as we were unable to find any corresponding documents either in the victims’ register or in the archives available to us. In 32 cases we were contacted by women, mostly from the third generation (i.e., grandnieces, grandnephews, grandsons, and granddaughters) and usually by mail. In three cases we were able to support the family in their application to the National Fund of the Republic of Austria for Victims of National Socialism (www.nationalfonds.org). In most cases, the requests were from Austrians, one came from the USA, and three were from Germany.

The main reasons for the requests had to do with the desire to reconstruct family histories, or were related of psychotherapy, study, or genealogic studies. In only six cases did the reason relate to a feared link to mental illness within the family. Another often mentioned factor was time: there was little time left to speak with elderly relatives, to benefit from their knowledge and memories.

Our concern was primarily therapeutic rather than a research-oriented, in the knowledge that often, unresolved traumas in these families can impede the mourning process via transgenerational transference. We were able to identify and confirm many phenomena and models of the transgenerational transmission of traumatic experiences. Our objective was to support those relatives who wanted to break through the “Conspiracy of Silence” and to reactivate the impossible or paused mourning process. Our aim was to interrupt the transgenerational transmission, which has psychodynamic, sociocultural, systemic, and biological routes (Glaesmer et al., 2011).

Feelings of guilt and shame and fear, harbored by individuals as well as of families and society, are defended individually or collectively. All the defense mechanisms of in-depth psychological teachings can be found: repression, denial, projection. However, the events, the feelings, and the inner-psychic processes associated with them are not lost, but stored in the individual as well as in collective memory, often unconscious and still unresolved. The unresolved and interrupted mourning process is passed down to the next generation by a different kind of transmission. The progress and maturation of the individual and social groups, however, depend on the processing of these traumas.

The relevant documents were not handed to the relatives to digest alone, but discussed with the relatives. This was because families are often overwhelmed by pain and grief, or because the files might include completely surprising, painful details that are difficult to understand and absorb. The question of heritability always comes up. The potential transgenerational transmission of psychological structures leads to great consternation, even in subsequent generations. Family chronicles are (re)written, cross-generational walls of silence are torn down. Most intense are the conversations with the children of the victims. Their childhood was marked not only by the mental illness of their parents, but ultimately by their absence and killing, compounded by the subsequent taboo and stigmatization. Going to places of remembrance to mourn, and further contact—also in written form—can help here to alleviate somewhat the pain that often surges violently. The Carinthian Regional Health Insurance Fund offers free psychotherapy at the Aspis Association to the descendants of Nazi victims. We are available for further discussions or inquiries by telephone or by mail. After processing is completed, the files are returned to the National Archives. A copy of the requested files remains in-house.

What follows is a quote from a letter sent by one of the contacts supported in this process:

You have taken a great dark uncertainty from us through your work. Even though the fate of my great-grandmother hurts us very much, we now have certainty, a place and a date to mourn. You have given us the feeling of having “found” my great-grandmother again.

This work is done “because violence loses its destructive consequences (also for the next generations) when it is transferred from a taboo topic to a reflexively workable topic within the collective memory of a society” (Loch, 2019).
